# Protocol for studying cough frequency in people with pulmonary tuberculosis

**DOI:** 10.1136/bmjopen-2015-010365

**Published:** 2016-04-22

**Authors:** Alvaro Proaño, Marjory A Bravard, Brian H Tracey, José W López, German Comina, Mirko Zimic, Jorge Coronel, Gwenyth O'Neill Lee, Luz Caviedes, Jose Luis Cabrera, Antonio Salas, Eduardo Ticona, Daniela E Kirwan, Jon S Friedland, Carlton A Evans, David A Moore, Robert H Gilman, Tuberculosis Working Group in Peru

**Affiliations:** 1 Facultad de Medicina ‘Alberto Hurtado’, Universidad Peruana Cayetano Heredia, Lima, Perú; 2 Asociación Benéfica PRISMA, Lima, Perú; 3 Department of General Internal Medicine, Massachusetts General Hospital, Boston, Massachusetts, USA; 4 Innovation For Health And Development (IFHAD), Laboratory of Research and Development, Universidad Peruana Cayetano Heredia, Lima, Peru; 5 Department of Electrical and Computer Engineering, Tufts University, Medford, Massachusetts, USA; 6 Instituto Nacional de Salud del Niño San Borja, Lima, Perú; 7 Laboratorio de Bioinformática y Biología Molecular, Facultad de Ciencias y Filosofía, Universidad Peruana Cayetano Heredia, Lima, Perú; 8 Department of Global Community Health and Behavioral Sciences, Tulane University, New Orleans, Louisiana, USA; 9 Escuela Profesional de Ingeniería Física, Facultad de Ciencias, Universidad Nacional de Ingeniería, Lima, Perú; 10 Laboratorio de Investigación en Enfermedades Infecciosas, Laboratorio de Investigación y Desarrollo, Facultad de Ciencias y Filosofía, Universidad Peruana Cayetano Heredia, Lima, Perú; 11 Servicio de Neumología, Hospital Nacional Alcides Carrión, Lima, Perú; 12 Servicio de Neumología, Hospital Nacional Dos de Mayo, Lima, Perú; 13 Facultad de Medicina, Universidad Nacional Mayor de San Marcos, Lima, Perú; 14 Servicio de Enfermedades Infecciosas y Tropicales, Hospital Nacional Dos de Mayo, Lima, Perú; 15 Infectious Diseases & Immunity, Imperial College London, London, UK; 16 Wellcome Trust Imperial College Centre for Global Health Research, London, UK; 17 TB Centre, London School of Hygiene and Tropical Medicine, London, UK; 18 Program in Global Disease Epidemiology and Control, Department of International Health, Bloomberg School of Public Health, Johns Hopkins University, Baltimore, Maryland, USA

**Keywords:** Cough, Monitoring

## Abstract

**Introduction:**

Cough is a key symptom of tuberculosis (TB) as well as the main cause of transmission. However, a recent literature review found that cough frequency (number of coughs per hour) in patients with TB has only been studied once, in 1969. The main aim of this study is to describe cough frequency patterns before and after the start of TB treatment and to determine baseline factors that affect cough frequency in these patients. Secondarily, we will evaluate the correlation between cough frequency and TB microbiological resolution.

**Methods:**

This study will select participants with culture confirmed TB from 2 tertiary hospitals in Lima, Peru. We estimated that a sample size of 107 patients was sufficient to detect clinically significant changes in cough frequency. Participants will initially be evaluated through questionnaires, radiology, microscopic observation drug susceptibility broth TB-culture, auramine smear microscopy and cough recordings. This cohort will be followed for the initial 60 days of anti-TB treatment, and throughout the study several microbiological samples as well as 24 h recordings will be collected. We will describe the variability of cough episodes and determine its association with baseline laboratory parameters of pulmonary TB. In addition, we will analyse the reduction of cough frequency in predicting TB cure, adjusted for potential confounders.

**Ethics and dissemination:**

Ethical approval has been obtained from the ethics committees at each participating hospital in Lima, Peru, Asociación Benéfica PRISMA in Lima, Peru, the Universidad Peruana Cayetano Heredia in Lima, Peru and Johns Hopkins University in Baltimore, USA. We aim to publish and disseminate our findings in peer-reviewed journals. We also expect to create and maintain an online repository for TB cough sounds as well as the statistical analysis employed.

**Strengths and limitations of this study**

- The algorithm employed in this project has been validated specifically for patients with pulmonary tuberculosis (TB), which enables us to use this algorithm in our patients.
- A strength of this project is that its results will reflect actual cough frequency episodes in pulmonary TB by utilising 24 h recordings in the patients' normal-day settings (traffic, dogs barking, etc). We expect that this will generate a novel method of evaluating cough in TB that can be used in real-world scenarios.
- Our study has the limitation that recordings have been processed through a semiautomated algorithm. To decrease time constraints, our long-time goal is to create a fully automated processing system. We anticipate that experience gained with semiautomated analysis will aid us in developing future algorithms.

## Introduction

Tuberculosis (TB) is an infectious disease, and was responsible for 9.6 million new cases and 1.5 million deaths in 2014.^1^ TB is transmitted in the air^2^ ^3^ and cough is the most important cause of transmission.^4^ Cough in people with pulmonary TB disease arises as a result of the inflammatory response to mycobacterial pulmonary infection. A reduction in cough is assumed to represent adequate response to treatment, and to result in decreased risk of spread of infection. Despite its crucial role in TB transmission, a recent literature review^5^ reported that cough frequency during TB therapy has not been studied since the work carried out by Loudon in the 1960s.^6^ ^7^ Thus, longitudinal cough frequency studies in TB are needed.

Loudon described cough frequency in 8 h overnight periods for 9 weeks. All sounds with amplitude and frequency consistent with possible cough events were recorded and then manually reviewed.^8^ His findings show a twofold reduction in the first 2 weeks of treatment, from a mean of 13.6 to 4.75 coughs/h.^7^ *Mycobacterium tuberculosis* colony forming units (CFU) also reduced significantly from 10^6^ at baseline to 10^3^ 2 weeks later.^9^ ^10^ This evidence led to the idea that drug-susceptible patients with TB become sufficiently non-infective by the second week of treatment that they no longer pose a risk to others. This and other evidence led to the often-used policy that 2 weeks was the necessary duration of respiratory isolation for newly diagnosed patients started on appropriate treatment. Current evidence^11^ and guidelines affirm this position;^12–14^ however, this 2-week policy has been criticised.^15^ ^16^ Our group has shown that drug-susceptible patients with TB remain sputum culture positive for longer.^17^ ^18^ Most importantly, the assumption that patients with TB are no longer coughing at 2 weeks has never been corroborated.

The 2015 CHEST guidelines recommend acoustic parameters to evaluate the frequency of cough.^19^ In order to ensure accurate measurement, it is important to use a standardised method such as automated cough counting with a validated algorithm. Despite the recently growing literature on this topic, these methods are principally being used in the field of non-infectious chronic disease.^20–25^ While algorithms for cough counting have been validated,^26–30^ our research protocol appears to be the first to do so specifically in patients with pulmonary TB.^31^ ^32^

To address this knowledge gap, we have developed the Cayetano Cough Monitor (CayeCoM) and here describe a protocol for it to be used to study cough frequency in patients with pulmonary TB.

## Methods

### Study objectives

*The primary objective of this study* is to describe cough frequency patterns in adults with pulmonary TB before and after treatment initiation.

*The second objective of this study is* to determine baseline characteristics that correlate with cough frequency, such as patient demographics, radiological findings, presence of multidrug-resistant TB (MDR-TB) and HIV status.

*The third objective of this study* is to test for an association between changes in cough frequency and microbiological resolution of TB disease during therapy.

### Study design

This prospective cohort study will follow adult patients with pulmonary TB throughout their treatment period in Lima, Peru.

Participants with a confirmed or suspected diagnosis of active pulmonary TB will be referred to our study team. After obtaining written informed consent, we will record coughs prior to initiation of TB treatment. Participants will provide us with early morning sputum samples that will be tested for active pulmonary TB disease by testing at least one sputum sample using the microscopic observation drug susceptibility (MODS) broth culture assay^33–35^ and auramine smear microscopy to assess the bacillary load.

**Figure 1:**
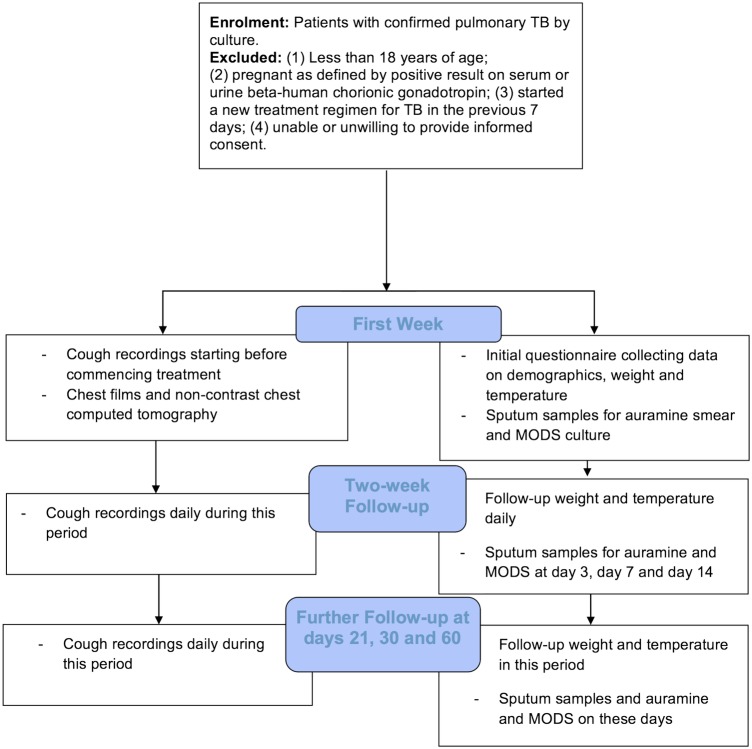
Flow diagram for CayeCoM study. CayeCoM, Cayetano Cough Monitor; MODS, microscopic observation drug susceptibility; TB, tuberculosis.

Patients in whom the pulmonary TB diagnosis is confirmed by MODS will receive treatment delivered by the National TB Programme as per standard practice.^36^ Figure 1 summarises the data to be collected at baseline and during the 60 days of follow-up.

### Study sites

Peru has one of the highest TB incidence rates in the Americas.^37^ More than one-third of the incident TB cases in the Andean region are from Peru. With respect to rates of MDR-TB and extensively drug-resistant (XDR) TB, Peru ranks first in all of the Americas. However, under-reporting in the region may contribute to Peru's over-representation, as shown in the latest Pan American Health Organisation (PAHO) report.^37^

Within Peru, Lima and its metropolitan area account for most cases of MDR-TB and XDR-TB.^38^ Thus, we will recruit patients from two hospitals: Hospital Nacional Dos de Mayo (HNDM), located in the historic centre of Lima; and Hospital Nacional Daniel Alcides Carrión, located in Callao and which belongs to Lima's metropolitan area.

Our main site, HNDM, is a 650-bed teaching and public national tertiary referral hospital run by the Peruvian Ministry of Health (MINSA). It provides services to the poor population from the surrounding inner city area. HNDM is the only hospital in Peru with a negative pressure ward available for patients with TB. Our secondary site is another tertiary referral hospital run by MINSA, Hospital Nacional Daniel Alcides Carrión. This 462-bed teaching health facility lies in the Callao region.

### Study population

The infectious disease and pulmonary physicians will refer participants to the research team. Criteria for referral are suspicion of active pulmonary TB or a confirmed case of active pulmonary TB which has not yet started treatment. Active pulmonary TB is defined by a positive MODS culture result. Participants will be excluded if they were <18 years of age, pregnant, have started a new treatment regimen for TB within the past 7 days, or are unable or unwilling to provide informed consent. If a patient changes the treatment regimen, for example, due to treatment failure or to an adverse drug reaction, this would also be considered as a new regimen. Pregnancy is defined by a positive result on the serum or urine β-human chorionic gonadotropin (β-hCG) assay.

### Outcomes and case definitions

The primary outcomes for this study are cough frequency and microbiological data from serial sputum samples. Cough frequency is defined as the number of cough episodes, or cough epochs, within a time period. Cough epochs are defined as cough events that are within a 2 s period frame.^32^

Regarding microbiological data, participants will be entered into the study if they have a positive culture result. Treatment regimens will be adjusted as needed by the treating team based on the results of the MODS drug-susceptibility testing from their sputum. Our study team will not be involved in the treatment regimen selection.

Sputum smear conversion is defined as three consecutive smear-negative results, collected at least 8 h apart after initial smear positivity at diagnosis.^12^ Culture conversion is defined as two consecutive negative culture results, taken at least 30 days apart. This last definition is the one used in the MINSA^36^ and is recommended by the WHO.^39^ The date of conversion will be considered as the date of the first negative sputum smear or culture contributing to conversion.

Secondary outcomes include weight, temperature and radiological characteristics. When possible, radiological interpretation data from chest films and thoracic CT scans will be obtained. Chest X-ray films (CXR) provide a high negative predictive value for the presence of active TB,^40^ but CXR might be normal when in fact there is parenchymal disease.^41^ More specifically, CT scans correctly determine pulmonary TB cases in 91% of cases and CXR in only 49% of cases.^41–44^ In addition, CT scans provide higher sensitivity for the detection of lymphadenopathy, early bronchogenic spread and to evaluate cavitation and disease activity.^44^

### Sample size

In a pilot study, we estimated that the frequency of cough in patients with TB before receiving treatment is approximately 327 coughs during a 24 h period with an SD of approximately 50. A sample size of 97 patients would enable us to detect a conservative decrease in the mean number of coughs in the 24 h period of at least 45 coughs after 2 weeks of treatment, with a 5% type I error probability and 80% power.

Under the hypothesis that patients with TB before treatment experience a high cough frequency, we hypothesise that after 2 weeks of anti-TB treatment, there will be a clinical response accompanied by a significant reduction in cough frequency. Response is defined as at least a twofold reduction in cough frequency, which was previously shown to occur within the first 2 weeks of treatment.^7^ For power calculations, it is assumed that all participants will eventually respond to treatment, according to our definition of response, and that once cough frequency has reduced in an individual it will not rise again. We assume that after the 2 weeks of treatment approximately 10% of patients would maintain a high frequency of cough. Thus, a sample size of 97 patients will allow us to detect an OR of at least 3.2 for the risk of patients not responding to TB treatment in 2 weeks of therapy, under a 95% significance and 80% power. An additional 10% of patients will be recruited to correct for patients who do not complete all of the study procedures. Thus, we will aim to recruit a total of 107 patients.

### Study organisation

The Asociación Benéfica (A.B.) PRISMA and Universidad Peruana Cayetano Heredia in Lima, Peru will provide local administrative oversight. Overseas, oversight will be conducted by Johns Hopkins University in Baltimore, Maryland, USA.

In Lima, the Pampas office of A.B. PRISMA will provide operations and logistic support for fieldwork. An additional collaborating signal processing team will be based locally in the Universidad Nacional de Ingeniería, Lima, Peru, as well as at Tufts University, Massachusetts, USA.

Our collaborating biostatisticians are based at Tufts University, Tulane University and Universidad Peruana Cayetano Heredia, Lima, Peru. All investigators are involved in protocol design and technical support and will remain involved in the ongoing analyses.

### Personnel, training and logistics

Nurses have been trained by study staff to obtain sputum samples in a best-practice fashion based on previous work,^45^ ^46^ and to operate and troubleshoot all recorder devices, memory cards and battery packs. We will adhere to recommended infection prevention and control practices for TB to reduce biorisk in healthcare professionals and patients.^47^ Written informed consent is required prior to research participation. At the time of enrolment, participants will follow the procedures outlined in figure 1.

Participants with active pulmonary TB will be followed throughout their TB treatment. After the identification of active pulmonary TB and on the basis of convenience, participants who consent will undergo CXR and a non-contrast thoracic CT scan.

The first day of a new TB treatment regimen is defined as ‘day 0’. An initial questionnaire will be completed on that day (see online supplementary file 1). It should be mentioned that we used a 5-level ordinal scale instead of 10 to make it simpler for our interviewees. We have found it easier in this setting for research participants to interpret 5-levels each with defining words (never, little, much, almost always and always) rather than 10. This questionnaire is similar to the one that was employed in a previous study.^48^ Baseline cough frequency will be obtained by performing an audio recording of the patients before they obtain their microbiological results, which is usually a few days prior to treatment initiation. Hence, participants will be recorded from at least one day prior to treatment and throughout their first two weeks of treatment. They will subsequently be recorded for 24 h on or around days 21, 30 and 60 of treatment, although up to two days’ date deviation for Sundays and public holidays will be allowed.

10.1136/bmjopen-2015-010365.supp1 Supplementary data

Recordings will start at 09:00 and will be as continuous as possible. Occasionally, incomplete recordings could be obtained due to malfunction of equipment or patient non-compliance. On the recording days, clinical data will be gathered, including: weight, temperature and sputum samples for smear and MODS results. The number of days to culture positivity in the MODS liquid culture assay will be recorded in order to assess the microbiological burden in the patients' samples, based on prior work done with a similar technique.^49^

### Audio recording

**Figure 2:**
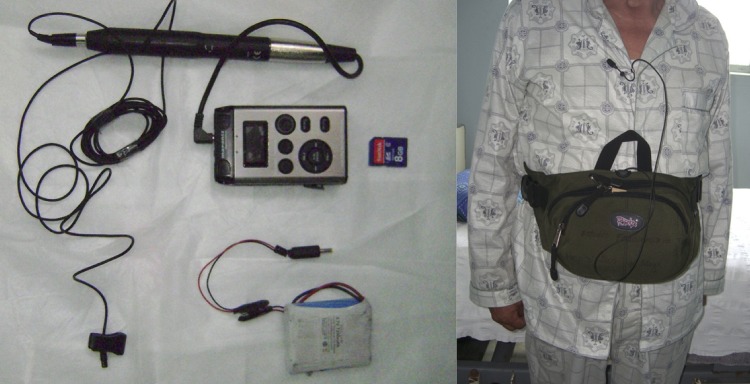
Picture of the Cayetano Cough Monitor (CayeCoM).

Design of the audio recording equipment, the CayeCoM device, builds on previous chronic cough ambulatory audio recordings.^27^ ^50^ ^51^ The CayeCoM device is a Marantz PMD 620 professional handheld recorder, using an Audio-Technica AT899 sub-mini microphone with an AT8537 microphone power module. The microphone will be attached at the patient's lapel as shown in figure 2. The recorder is adapted to work with an external lithium battery supply (Enix Energies 800040) to enable continuous 24 h recordings. The audio is recorded onto a SanDisk SDHC 8 GB card, at a sample rate of 48 kHz, encoding 64 kbps in mono in MP3 format. The audio equipment is kept inside a basic pack connected to a lapel microphone. Batteries and SD cards will be exchanged daily by the study nurses. In pilot research, participants tolerated the audio equipment well, wearing them 24 h a day and taking them off only to bathe.

### Processing of audio recordings

The recorded signals will be analysed after all patient recordings are completed. For cough analysis, software developed by our group and previously described in detail will be used.^31^ ^32^ Thus, we provide only a brief review here and refer interested readers to our previous publications.

Briefly, cough recordings will be analysed using a two-step algorithm: first, event detection, followed by event classification into cough versus non-cough. Detection of acoustic events will be based on the signal energy proportional to the square of the voltage of the signal. An acoustic event will detect if the signal energy exhibited a rapid increase above a time-varying baseline estimate of ambient noise. The next stage of processing seeks to classify detected events. Here, the spectral features of each time frame in the acoustic event are characterised using Mel-frequency cepstral coefficients and their derivatives. As described in detail elsewhere,^32^ a training data set will be used to develop a classifier based on the sequential minimal optimisation (SMO) algorithm. On the basis of classifier outputs, each acoustic event will be marked as ‘cough’ or ‘not-cough’.

Isolated cough events will be automatically combined into cough epochs, or bursts of closely spaced individual coughs, following previous research.^52^ We will employ a definition of cough epochs, as defined in the ‘Outcomes and case definitions’ section above. Note that within the cough literature, a variety of metrics are available for describing a cough, and there is no clear evidence as to which of them are most clinically meaningful. We have previously published a review and discussion of these various metrics (number of individual coughs, number of cough bouts or epochs, number of 1 s periods containing cough, etc).^32^

We will employ a semiautomated approach in which cough epochs that are automatically detected will then be manually reviewed to eliminate false positives. This is necessary as our recordings will be made in very noisy environments (outside clinical settings) and false detection rates for a fully automated system remain high. For this study, a simple graphical user interface will be constructed to allow nurses to review automatically detected epochs, enabling them to listen to each as often as needed, and then to either accept or reject the detected cough. Thus, the review of automatically detected coughs acts to eliminate algorithmic false-positive coughs.

*Validation*: The approach described in the above paragraphs was previously validated using as the gold standard a fully manual review of 60 files (15 participants, 4 randomly selected time periods per participant) in which two nurses listened to all files in their entirety.^32^ Since nurses only manually marked the start of each cough, validation was compared on the basis of the epoch definition described above. The semiautomated approach described above gave 75.5% sensitivity in detecting coughs (a true-positive rate of 6.8/h) with an average false-positive rate of 0.5/h.^32^ While the semiautomated approach does require time for human review, the initial automated step will remove the large majority of possible events. Thus, on average, review time is reduced by nearly two orders of magnitude compared with a fully manual review in which the entire recording is reviewed. We will also maintain the privacy of participants, as non-cough events, such as conversation, will never be reviewed by the human ear.

### Microbiology

The microbiological tests will be carried out in a Biosafety Containment Level 3 research laboratory situated within Universidad Peruana Cayetano Heredia in Lima, Peru. The sputum samples will be digested and decontaminated by the standard NaOH-n-acetyl cysteine method.^53^ For smear microscopy, an aliquot of 100 µL is stained with Auramine O and examined with ×400 magnification. Results are determined as negative, paucibacillary (1–19 acid-fast bacilli (AFB) visualised in 40 fields), 1+ (20–199 AFB visualised in 40 fields), 2+ (5–50 AFB per field) and 3+ (>50 AFB per field). Culture and MODS susceptibility testing will be performed with the remaining samples, according to standard protocols.^33–35^

### Radiology

Radiological information will be gathered when possible on a convenience basis. Priority will be given to CT scans, since they have been shown to be more sensitive in general.^44^ A previous study determined that the sensitivity for the prediction of active TB through CT scans was 96%, whereas for CXR it was merely 48%.^54^ Films will be read by a local radiologist and a US board-certified radiologist blinded to the patient's demographics and outcomes. They will provide an interpretation that is standardised as per our study protocol to describe radiological findings including cavitation, consolidation, lymphadenopathy and effusions (see online supplementary file 2). We will explore whether these radiological findings are predictive of microbiological burden and cough frequency.

10.1136/bmjopen-2015-010365.supp2 Supplementary data

Cavitations will be further described by the size, location, presence or absence of an air–fluid level, and cavity wall thickness based on prior work that shows the relevance of these findings to pulmonary TB and, most importantly, to infectivity.^7^ ^55–58^ It is therefore important to determine cavitations, and as Im *et al*^55^ have shown, CT correctly identifies cavitations in 58% of cases, whereas CXR only identifies 22%.

### Statistical methodology and analysis

All questionnaire data will be double digitised from paper forms using Visual FoxPro 9 Service Pack 2 (Microsoft Corp. Redmond, Washington, USA) and microbiological data will be double entered using Microsoft Access 2010 (Microsoft Corp. Redmond, Washington, USA). These two data sets will be cross-compared for validity and errors. From these data, descriptive statistics will be tabulated and graphed.

Cough analysis processing results will be stored as Matlab (Mathworks, Inc, Natick, Massachusetts, USA) files containing information regarding each event and its timestamp. Algorithmically detected coughs will be annotated in the files. After manual review, isolated cough events will be grouped into cough epochs, or bursts of closely spaced individual coughs within 2 s, following published work on cough evaluation.^52^

*For the first study objective of describing cough frequency*, cough epochs will be plotted throughout the day, and cough frequency will be summarised as the frequency of cough epochs per hour. Positively skewed cough data may be log-transformed to facilitate data visualisation and analysis. *To address the second study objective*, correlation of characteristics with cough frequency, we will use generalised estimating equations (GEE) based Poisson or negative binomial regression with baseline microbiological status, and trigonometric (sine/cosine) terms to model circadian periodicity, as the independent variables. In addition, a multiple logistic regression in a longitudinal generalised linear model (GLM) framework analysis will evaluate a function of sputum bacillary load and with cough frequency that we propose as a potential predictor of TB transmissibility. In all cases, we will correct for outliers, and nested models will be compared using the likelihood ratio test. We will also consider variables such as gender, HIV status, drug resistance and history of TB in our analysis, either by stratifying or by adjusting for these variables in our models.

To test the association between cough frequency and microbiological resolution of TB disease associated with the third aim of this study, time-to-event survival analyses where the outcomes of interest are sputum smear conversion, and culture conversion, as defined above, and the primary predictors of interest are cough frequency at baseline, during treatment, and time to twofold reduction in cough frequency. In addition, secondary analyses of weight, temperature and radiological characteristics will be conducted using GLMs and GEE logistic regression as appropriate.

### Dissemination

Written informed consent will be obtained from all participants. Test results will be delivered by telephone or at subsequent visits at which time a team physician or nurse will be able to explain the results to the study participants. TB treatment remains the responsibility of the medical staff in charge and the National TB Programme. We aim to publish and disseminate our results once the project is complete. We also expect to create and maintain an online repository for TB cough sounds as well as the statistical analysis employed.

## Discussion

We will determine cough frequency before and during anti-TB treatment using the CayeCoM device. We will identify baseline predictors of cough frequency during TB treatment and evaluate the correlation between change in cough frequency and microbiological resolution.

The medical literature currently lacks information about cough frequency in TB. As recently noted by Turner and Bothamley,^5^ cough frequency in patients undergoing TB treatment has only been studied once, almost half a century ago.^6^ ^7^ This previous study has the limitation of only being conducted within an 8 h period, overnight, and thus there is no information on daytime coughing or the effect of the diurnal rhythm on cough. A similar study^58^ demonstrated that the severity of cough and pathological chest X-ray findings were associated with higher levels of TB transmission. However, their study did not measure cough frequency but instead focused on a participantive characteristic: cough severity. It should be noted that to assess cough frequency, one must utilise objective acoustic parameters, since self-reported cough is unreliable.^19^ As reported in abstract form, the objective acoustic Leicester Cough Monitor (LCM) has been used to evaluate 24 h cough recordings in patients with pulmonary TB before starting treatment, showing that cough frequency is reduced at night.^59^ This further justifies re-evaluation of Loudon's overnight study.

Our project has several strengths and limitations. An important strength is the generation of 24 h cough recordings, which will provide lengthy recordings, will enable evaluation of cough patterns at different times of day, and also have the benefit of being recorded during a normal day in real-world settings where we expect our device to be used in the future. Normal day recordings are confounded by background noise, which is a challenge for analysis of cough recordings, considering that traffic and environmental noise (such as dogs barking, music and television) may generate noises similar to cough. To diminish this effect, we have incorporated a time-varying estimate of the noise background as well as a data quality control. Having a semiautomated algorithm is a limitation, since it requires time and human input, as well as a strength since the human ear is the gold standard for determining the characteristic sound of cough. Similar to Loudon and Romans'^8^ proposal, our algorithm will help to screen and reduce the length of the recordings to ∼5% of their original length, without affecting sensitivity and improving specificity.^32^ In the long term, we aim to improve our algorithm (ie, fully automated processing), and we anticipate that experience gained with semiautomated analysis will aid us in this process. In addition, we are now developing second-generation devices where the validity is improved by employing accelerometers. This study is limited by restriction to only non-pregnant adults because this is the population for which the algorithm has been validated. However, future research is planned to include these important vulnerable populations.

CayeCoM has been validated for 24 h recordings,^32^ whereas PulmoTrack (PulmoTrack-CC, KarmelSonix, Haifa, Israel) was validated for 25 min^29^ and the Hull Automatic Cough Counter for 1 h recordings.^27^ Other systems have also validated their algorithms for 24 h recordings, such as the LCM,^28^ ^60^ VitaloJAK^30^ and the LifeShirt System.^26^ However, in contrast to our study, none of these algorithms have been validated either for pulmonary TB or within real-life settings (eg, traffic). We expect that this project will generate a novel method to evaluate treatment response. In future studies, we intend to better assess infectiousness by additionally quantifying TB in cough-generated aerosols.

Cough frequency should provide additional information regarding the evolution of the patients' medical condition. If a correlation with bacteriological treatment response is demonstrated, then this would have the potential to contribute to patient management without relying on a laboratory in adult patients with pulmonary TB. However, we should be careful when monitoring patients with TB since some may worsen after an initial positive response to therapy. It could assist with decisions regarding the need for the ongoing respiratory isolation of patients, treatment duration and identification of patients with treatment failure who may need modification of their treatment regimens. The device also has the potential to be used remotely, as in telemedicine. This is potentially important in a country such as Peru, where the majority of doctors live in the capital, leaving most of the country without a physician in their region. Cough monitoring devices seem challenging; however, we believe that this is the first step towards telemedicine in cough-TB. In Peru, many rural areas do not have facilities for laboratory diagnosis, but have at least one physician or healthcare professional. They may be trained in placing these devices. We are also working on making devices smaller, cheaper and easier to use.
