# Supplementary material for: Protocol for studying cough frequency in people with pulmonary tuberculosis
Source: BMJ Open. 2016 Apr 22;6(4):e010365. doi: 10.1136/bmjopen-2015-010365 (PMC4853990; doi:10.1136/bmjopen-2015-010365)
Supplement: Supplementary data [file bmjopen-2015-010365supp1.pdf]

Código del paciente: ________________
Fecha de Entrevista: ___/___/____ Iniciales del Entrevistador: __________

Entrevistador: ____

Cuestionario Inicial Para Todos Los Participantes:

Datos Demográficos:

1. Edad: _____
2. Sexo: ______

Antecedentes:

3. ¿Cuántas personas normalmente duermen en casa?: _______ Personas

4. ¿Cuántas habitaciones hay en su hogar? (sin contar baño, pasadizo, cocina, depósito, garaje): ______ habitaciones.

5. ¿Cuál es el ingreso mensual de la vivienda? S/.__________________

6. ¿Cuánto gasta su familia en alimentación cada semana? S/.__________________

7. ¿Cuántas personas en su vivienda comen de esos alimentos que compran semanalmente? ____________ Personas

8. ¿Cuántas veces en el ultimo mes usted se ha acostado con bastante hambre porque no había comida en casa? ________ días

Historia de Tuberculosis:
La tuberculosis es una enfermedad que se trata con varios antibióticos a la vez, y cuyo tratamiento dura varios meses.

9. ¿Ha sido diagnosticado con TBC anteriormente?
   a. Si
   b. No → *Pase a la pregunta 15*
   c. NS

10. ¿Cuántas veces? ______________________

11. Si recibió tratamiento para la TBC, ¿dónde recibió la mayor parte del tratamiento?
    a. NA
    b. Mismo distrito
    c. Otro distrito
    d. Otra ciudad
    e. Otro país

12. Si recibió tratamiento para la TBC ¿por cuantos meses en total lo tomó? _____

13. Si recibió tratamiento previo, en que esquema estaba (lo mas recién): - ______________

14. Si recibió tratamiento para la TBC ¿cumplió con el tratamiento previo?
    a. NA
    b. Si
    c. No→ ______________________
    d. NS

Factores de Riesgo

15. ¿Ha compartido un cuarto con alguien que haya tenido TBC comprobada?
    a. Si, y esta persona también tenía una tos persistente
    b. Si, pero esta persona concurrente no tenía una tos persistente
    c. No → *Pase a la pregunta 18*
    d. NS

16. ¿Dónde compartió este ambiente con alguien infectado con TBC?
    a. NA
    b. Trabajo
    c. Casa
    d. Hospital
    e. Otro:______________________

17. ¿Por cuantos días compartió este ambiente con la persona con TBC comprobada? ______________

18. Aparte de usted, ¿alguien mas en casa esta actualmente recibiendo medicinas para la TBC?
    a. Si → Quien: ___________________________
    b. No
    c. NS

Creencias y Conocimiento de la Enfermedad

19. ¿Dónde escuchó de la TBC por primera vez?
    a. Familia
    b. Amigos
    c. Colegio
    d. Puesto de Salud
    e. TV
    f. Radio
    g. NS

20. ¿Puede alguien con TBC y tos infectar a sus familiares?
    a. Si
    b. No
    c. NS

21. ¿Qué tan contagiosa cree que es la TBC?
    a. Nada
    b. Poquito
    c. Mucho
    d. Bastante
    e. Lo mas

22. ¿Qué tan seria cree que es la TBC?
    a. Nada
    b. Poquito
    c. Mucho
    d. Bastante
    e. Lo mas

23. En general, ¿qué puede hacer uno por si mismo para protegerse de contraer la TBC? [Marcar el factor más importante, no sugerir repuestas].
    a. Vacunarse
    b. Comer
    c. Dormir bien, descansar
    d. Vivir una vida ordenada
    e. Mantenerse alejado de la gente con TBC
    f. Educarse
    g. Otro: ___________________
    h. NS

¿Cuales son los síntomas de la TBC?
[Marcar los factores, no sugerir repuestas].

24. Tos mencionada
    Si☐ No☐
25. Hemoptysis mencionada
    Si☐ No☐
26. Fiebre mencionada
    Si☐ No☐
27. Baja de peso mencionada
    Si☐ No☐
28. Fatiga, decaimiento mencionada
    Si☐ No☐
29. Palidez, un cierto semblante mencionada Si☐ No☐

30. ¿Qué debe hacer una persona con TBC para mejorarse? [Marcar el factor más importante, no sugerir repuestas].
    a. Tomar sus medicinas, asistir a controles
    b. Comer mas, comer mejor
    c. Descansar
    d. Tener fe
    e. Abrigarse
    f. Otro: ___________________
    g. NS

31. ¿Cómo puede hacer una persona con TBC para no contagiar la TBC a otros? [Marcar el factor más importante, no sugerir repuestas].
    a. Cubrirse la boca al toser
    b. Quedarase en casa, mantenerse alejado
    c. Seguir el tratamiento
    d. Separar cubiertos
    e. Otro: ___________________
    f. NS

32. ¿Se puede curar la TBC?
    a. Siempre
    b. Normalmente sí
    c. A veces
    d. Raramente
    e. Nunca

Instrucciones: Marque una X sobre la línea la posición que escoges.
Ejemplo: ----------- ------------

33. Con que frecuencia esta tosiendo hoy, en un promedio de 24 horas?

0	1	2	3	4	5
nunca	poquito	mucho		casi siempre	

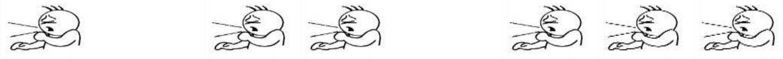

Instrucciones: Marque una X, en el cuadrado, todas las alternativas que corresponden.
Ejemplo: ☒

34. Que indicadores utilizó para escoger a que posición poner sus X?
    ☐ Los números: 1, 2, 3, 4, 5
    ☐ Las palabras: nunca, poquito, mucho, casi siempre
    ☐ Las figuras: 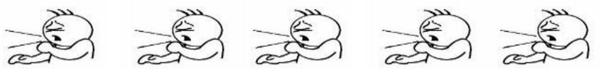

Código del paciente: ________________
Fecha de Entrevista: ___/___/____   Iniciales del Entrevistador: __________

Instrucciones: Marque con X la alternativa que corresponde.
Ejemplo: ☒

35. ¿Comparado con hace tres días, tiene más frecuencia de tos el día de hoy?
    a. ☐Tosiendo ahora menos
    b. ☐Tosiendo ahora igual
    c. ☐Tosiendo ahora más

36. El día de hoy, ¿ha tenido *solo tos, tos con flema o solo flema?*
    a. ☐Solo tos
    b. ☐Tos con flema
    c. ☐Solo flema

37. Si tiene tos, ¿a que hora en el día tiene más frecuencia: *mañana, tarde o noche*?
    a. ☐Mañana
    b. ☐Tarde
    c. ☐Noche

38. Qué tan frecuentemente tose?
    a. ☐Cada pocos segundos
    b. ☐Cada pocos minutos
    c. ☐Cada pocas horas
    d. ☐No tose

Instrucciones: Marque el numero de días en total que tiene los siguientes síntomas desde que se enfermó. Marque 0 si ningún día.

39. ¿Cuantos días usted ha tenido los siguientes síntomas?
    a. Tos seca ______
    b. Tos con flema ______
    c. Tos con sangre ______
    d. Fiebre ______
    e. Falta de aire ______
    f. Perdida de peso ______
    g. Cansancio o decaimiento ______
    h. Sudor nocturno ______
    i. Falta de apetito ______

Instrucciones: Responde a la pregunta en el lugar indicado.

40. Actualmente, ¿como se siente? (0 = mal, 10 = bien): _____

41. Cuando tiene tos, ¿cuantas toses tiene por hora? _____

42. Preguntas sobre VIH

		Manifiesto del paciente	Historia clínica
a.	Usted ha sido diagnosticado por VIH?	________	________
b.	Fecha de Diagnostico:	________	________
c.	Ultima Carga Viral:	________	________
d.	Fecha de ultima Carga Viral:	________	________
e.	Ultimo resultado CD4:	________	________
f.	Fecha de Ultimo resultado CD4:	________	________
g.	Tiempo tomanda TARGA (años)	________	________
