# Supplementary material for: Protocol for studying cough frequency in people with pulmonary tuberculosis
Source: BMJ Open. 2016 Apr 22;6(4):e010365. doi: 10.1136/bmjopen-2015-010365 (PMC4853990; doi:10.1136/bmjopen-2015-010365)
Supplement: Supplementary data [file bmjopen-2015-010365supp2.pdf]

CODIGO PACIENTE: ________________

Radiologic Interpretation Data Form

Date of read: ____

Radiologist Name: __________________________________

Radiologist Signature: ______________________________

Patient code: ____

Patient gender: ☐ male ☐ female

Patient age: ____

---

1. **Type of film:**
   ☐ AP ☐ PA ☐ Lateral from R ☐ Lateral from L ☐ Thoracic CT without contrast ☐ Other: ______________________________

2. Date of film: Day: ________ Month: ____________Year: ______________
3. Rotation:____
4. Adequacy of inhalation: _________________________________

	Site	a. Consolidation?	b. Cavitation?	c. Pneumatocele?	d. Atelectasis?
5	Right upper lobe	☐ yes ☐ no	☐ yes ☐ no	☐ yes ☐ no	☐ yes ☐ no
6	- anterior	☐ yes ☐ no	☐ yes ☐ no	☐ yes ☐ no	☐ yes ☐ no
7	- apical	☐ yes ☐ no	☐ yes ☐ no	☐ yes ☐ no	☐ yes ☐ no
8	- posterior	☐ yes ☐ no	☐ yes ☐ no	☐ yes ☐ no	☐ yes ☐ no
9	Right middle lobe	☐ yes ☐ no	☐ yes ☐ no	☐ yes ☐ no	☐ yes ☐ no
10	Right lower lobe	☐ yes ☐ no	☐ yes ☐ no	☐ yes ☐ no	☐ yes ☐ no
11	- superior	☐ yes ☐ no	☐ yes ☐ no	☐ yes ☐ no	☐ yes ☐ no
12	- basal	☐ yes ☐ no	☐ yes ☐ no	☐ yes ☐ no	☐ yes ☐ no
13	Left upper lobe	☐ yes ☐ no	☐ yes ☐ no	☐ yes ☐ no	☐ yes ☐ no
14	- anterior	☐ yes ☐ no	☐ yes ☐ no	☐ yes ☐ no	☐ yes ☐ no
15	- apical	☐ yes ☐ no	☐ yes ☐ no	☐ yes ☐ no	☐ yes ☐ no
16	- posterior	☐ yes ☐ no	☐ yes ☐ no	☐ yes ☐ no	☐ yes ☐ no
17	Lingula	☐ yes ☐ no	☐ yes ☐ no	☐ yes ☐ no	☐ yes ☐ no
18	Left lower lobe	☐ yes ☐ no	☐ yes ☐ no	☐ yes ☐ no	☐ yes ☐ no
19	- superior	☐ yes ☐ no	☐ yes ☐ no	☐ yes ☐ no	☐ yes ☐ no
20	- basal	☐ yes ☐ no	☐ yes ☐ no	☐ yes ☐ no	☐ yes ☐ no

21. Pleural effusion? ☐ yes ☐ no
left? ☐ small ☐ medium ☐ large
right? ☐ small ☐ medium ☐ large

CODIGO PACIENTE: ________________

22. Miliary spread? □ yes □ no
23. Pneumothorax? □ yes □ no
where and size: ____
24. Lymphadenopathy: □ yes □ no
which lymph nodes groups
□ hilar □ mediastinal
25. Pericardial effusion? □ yes □ no
left? □ small □ medium □ large
26. Bronchiectasis? □ yes □ no
where: ____
27. Fibrosis? □ yes □ no
where: ____
any retractions, deviations?____
____
____
28. Mediastinal thickening? ? □ yes □ no
29. Any tree-in-bud pattern? Where?____
____
____

30. Cavitation: For each cavity, please describe:
Cavity # 1:
location: ____
size (in mm) cephalic: ____
size (in mm) caudal: ____
size (in mm) anterior-posterior: ____
presence of air/ fluid level?: □ yes □ no
Cavity wall: □ think □ thick □smooth □nodular
Cavity Wall Thickness(in mm): ____

Cavity # 2:
location: ____
size (in mm) cephalic: ____
size (in mm) caudal: ____
size (in mm) anterior-posterior: ____
presence of air/ fluid level?: □ yes □ no
Cavity wall: □ think □ thick □smooth □nodular
Cavity Wall Thickness(in mm): ____

Cavity #3:
location: ____
size (in mm) cephalic: ____
size (in mm) caudal: ____

CODIGO PACIENTE: ________________

size (in mm) anterior-posterior: ____
presence of air/ fluid level?: □ yes □ no
Cavity wall: □ think □ thick □smooth □nodular
Cavity Wall Thickness(in mm): ____

Cavity # 4:
location: ____
size (in mm) cephalic: ____
size (in mm) caudal: ____
size (in mm) anterior-posterior: ____
presence of air/ fluid level?: □ yes □ no
Cavity wall: □ think □ thick □smooth □nodular
Cavity Wall Thickness(in mm): ____

Cavity # 5:
location: ____
size (in mm) cephalic: ____
size (in mm) caudal: ____
size (in mm) anterior-posterior: ____
presence of air/ fluid level?: □ yes □ no
Cavity wall: □ think □ thick □smooth □nodular
Cavity Wall Thickness(in mm): ____

More cavities? □ yes: please use another sheet to describe □ no

Other findings such as fractures, cardiac abnormalities, exudative / fibrotic densities, bronchogenic spread, mass-like lesions (calcified vs. non-calcified), please describe:

31. Normal film? □ yes □ no
